# The Role of Transglutaminase 2 in Cancer: An Update

**DOI:** 10.3390/ijms25052797

**Published:** 2024-02-28

**Authors:** Elisabetta Zaltron, Federica Vianello, Alessia Ruzza, Alberta Palazzo, Valentina Brillo, Ilaria Celotti, Matteo Scavezzon, Federica Rossin, Luigi Leanza, Filippo Severin

**Affiliations:** 1 Department of Biology, University of Padua, 35131 Padua, Italy; elisabetta.zaltron@unipd.it (E.Z.); federica.vianello.2@studenti.unipd.it (F.V.); alberta.palazzo@unipd.it (A.P.); valentina.brillo@phd.unipd.it (V.B.); ilaria.celotti@studenti.unipd.it (I.C.); matteo.scavezzon@studenti.unipd.it (M.S.); filippo.severin@unipd.it (F.S.); 2 Department of Biology, University of Rome “Tor Vergata”, 00133 Rome, Italy; federica.rossin@uniroma2.it

**Keywords:** transglutaminase 2, melanoma, breast cancer, ovarian cancer, colorectal cancer, leukemia, pancreatic cancer, lung cancer, microenvironment

## Abstract

Transglutaminase type 2 (TG2) is the most ubiquitously expressed and well characterized member of the transglutaminase family. It is a ubiquitous multifunctional enzyme implicated in the regulation of several cellular pathways that support the survival, death, and general homeostasis of eukaryotic cells. Due to its multiple localizations both inside and outside the cell, TG2 participates in the regulation of many crucial intracellular signaling cascades in a tissue- and cell-specific manner, making this enzyme an important player in disease development and progression. Moreover, TG2 is capable of modulating the tumor microenvironment, a process of dynamic tissue remodeling and biomechanical events, resulting in changes which influence tumor initiation, growth, and metastasis. Even if generally related to the Ca^2+^-dependent post-translational modification of proteins, a number of different biological functions have been ascribed to TG2, like those of a peptide isomerase, protein kinase, guanine nucleotide binder, and cytosolic–nuclear translocator. With respect to cancer, TG2′s role is controversial and highly debated; it has been described both as an anti- and pro-apoptotic factor and is linked to all the processes of tumorigenesis. However, numerous pieces of evidence support a tissue-specific role of TG2 so that it can assume both oncogenic and tumor-suppressive roles.

## 1. Introduction

Transglutaminases (TGs) comprise a family of nine multifunctional isoenzymes named TG1-7, blood coagulation factor XIII, and Band 4.2 [1,2,3,4,5,6]. First described by Waelsch in 1957, transglutaminase type 2 (TG2) is ubiquitously expressed and catalyzes calcium (Ca^2+^)-dependent post-translational protein transamidation.

**Figure 1:**
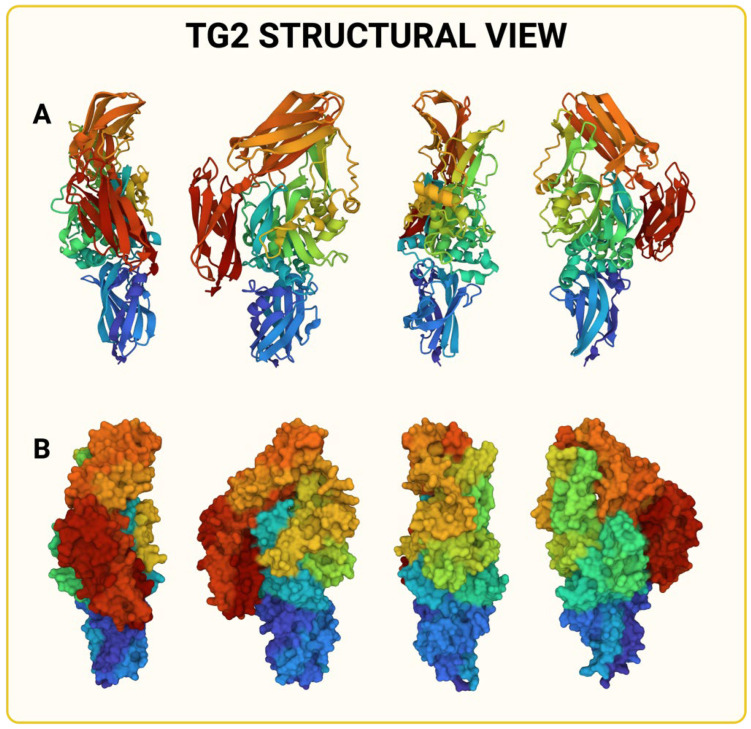
Representation of TG2′s structure from https://alphafold.ebi.ac.uk, accessed on 21 January 2024. (**A**) Tertiary structure of TG2. Different colors represent different domains. (**B**) TG2′s molecular surface. Different colors represent different domains.

Among the members of the TG family, TG2 is the most abundant and well-characterized transglutaminase in mammals [7] and is expressed in all cellular districts (nucleus, cytosol, and organelles), as well as in the extracellular environment [5,8,9]. While it retains strong sequence homology in its catalytic site [10] compared to other members of the transglutaminase family, TG2 has structural peculiarities that establish its uniqueness, making it a versatile protein [11] (see Figure 1). TG2 has four different protein domains, each of which is involved in various enzymatic activities. In addition to its primary transamidation function, TG2 has been demonstrated to possess Ca^2+^-independent GTPase, kinase, proteolytic, and scaffold activities [2,12,13].

TG2 can undergo a conformational change from a closed to an open state upon exposure to elevated intracellular Ca^2+^ concentrations triggered by various stimuli, and this is responsible for the activation of its cross-linking/transamidating activity [2,12]. TG2 has a great number of substrates inside cells, and the enzyme’s presence throughout cells allows for the formation of sub-cellular regions where Ca^2+^ levels rise locally, as observed during autophagy due to lysosomes’ release of Ca^2+^ [14]. Instead of Ca^2+^, TG2 can bind GTP, shifting to a closed signaling-active conformation which locks transamidase activity. In this closed state, TG2 functions as a GTPase [7,15]. TG2′s activity is further modulated by various cofactors, such as membrane lipids like sphingosyl phosphocholine, which can influence its susceptibility to Ca^2+^ levels [1,16]. In the closed state, TG2 cannot bind Ca^2+^; thus, GTP and Ca^2+^ binding and consequently GTPase and transamidase activity are mutually exclusive [1,2,11,17,18].

As stated above, TG2 has different substrates that can undergo post-translation modifications via different molecular mechanisms thanks to its multiple activities, such as those of transamidases, deamidases, GTPases, isopeptidases, adapters/scaffolds, protein disulfide isomerases, and kinases, hipusination regulation, and serotonilation [2,19].

Moreover, making the study of this enzyme complicated, TG2 activity is diverse depending on its subcellular localization. Therefore, TG2 is involved in different cellular processes like cell death, growth, adhesion, and differentiation, as well as inflammation, tissue remodeling, wound healing, and extracellular matrix (ECM) organization [6,20,21].

In addition, a structural role of TG2 was recently dissected [1]. Indeed, it has been demonstrated that TG2 contributes to the formation of mitochondria–ER contact sites by its interaction with Heat Shock Protein Family A (Hsp70) Member 9 (GRP75) and Inositol 1,4,5-Trisphosphate Receptor, Type 3 (IP3R); TG2 ablation leads to a wider and reduced number of contacts between the two organelles [22,23]. Moreover, its expression is important for supporting β-catenin accumulation inside the nucleus, where can regulate the transcription of Wnt-signaling-related genes, ultimately enhancing proliferation [17,24]. Finally, TG2 promotes cell proliferation, also modulating the signaling pathways of extracellular signal-regulated protein kinases 1 and 2 (ERK1/2) [17].

This transglutaminase also plays a fundamental role in ECM assembly. Indeed, it is linked to the regulation of some extracellular components which support cancer growth. TG2, stimulated by Epidermal Growth Factor (EGF) through the Ras and c-Jun kinase pathways, facilitates the motility and invasive capacity of tumor cells [25].

TG2 has been observed to exhibit protein disulfide isomerase (PDI) activity, a function linked to mitochondrial-dependent apoptosis [26,27]. This activity leads to the formation and breakage of disulfide bonds between cysteine residues [28]. Serine/threonine kinase activity phosphorylates specific substrates such as p53, histones H1-4, and retinoblastoma (Rb) protein, along with serotonylation and hypusination activities [29,30,31,32].

According to TG2′s multiple functions inside cells and in distinct tissues, it is clear that its deregulation is linked to numerous diseases, such as celiac disease, metabolic disorders, neurodegeneration, fibrosis, inflammation, and cancer, and its role is highly controversial and debated.

## 2. TG2 in Cancer

The pleiotropic nature of TG2 makes it an essential player in numerous cellular processes; it participates in cell growth and differentiation, adhesion and migration, autophagy, inflammation, tissue repair, fibrosis, extracellular matrix formation, proteostasis, apoptosis, angiogenesis, the EMT transition, and epigenetic modification [10,21,23,28,33,34]. The impairment of such a crucial enzyme that participates in several processes leads to different hallmarks of cancer, such as cancer progression, metastasis spread, cancer cell survival and invasion, and drug resistance. As reported in Table 1, TG2 has been shown to be involved in the resistance mechanisms of some of commonly used drugs.

**Figure 2:**
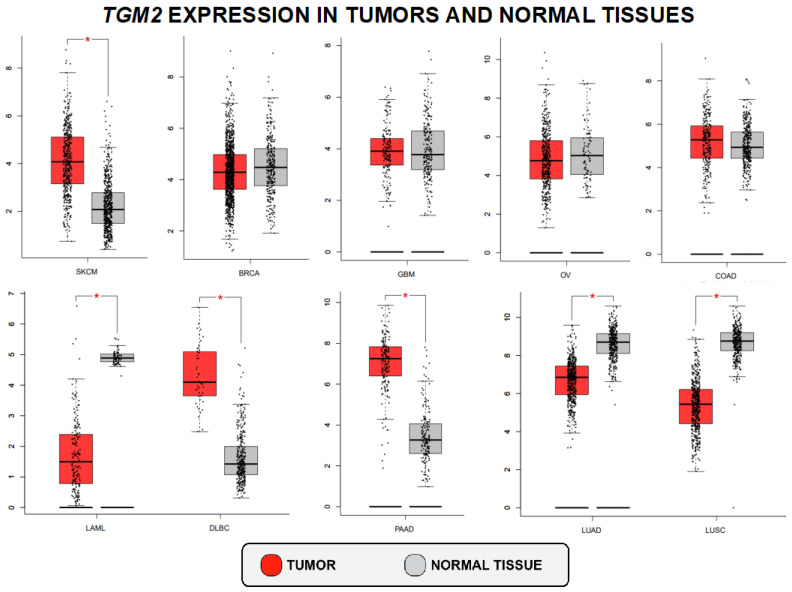
*TGM2* expression level in tumors vs. normal tissues on GEPIA. SKCM (skin cutaneous melanoma), BRCA (breast invasive carcinoma), GBM (glioblastoma multiforme), OV (ovarian serous cystadenocarcinoma), COAD (colon adenocarcinoma), LAML (acute myeloid leukemia), DLBC (lymphoid neoplasm diffuse large b-cell lymphoma), PAAD (pancreatic adenocarcinoma)0, and LUAD+LUSC (lung adenocarcinoma + lung squamous cell carcinoma). One-way ANOVA; *p*-value: * = <0.01.

TG2 expression was previously reported to be altered in several tumors [17,26,41,42,43]. An investigation of TG2 expression levels in patients affected by different types of cancers was conducted using the publicly available GEPIA (Gene Expression Profiling Interactive Analysis) database [27]. We compared *TGM2* mRNA expression levels in both tumor and normal tissues. Our analysis revealed significantly increased expression levels of the *TGM2* gene in tumor samples compared to controls for melanoma, diffuse large B-cell lymphoma, and pancreatic adenocarcinoma. Conversely, *TGM2* mRNA levels showed an opposite trend in acute myeloid leukemia and lung adenocarcinomas. Finally, *TGM2* did not exhibit statistically significant differences in breast carcinoma, glioblastoma, ovarian cancer, and colon adenocarcinoma (Figure 2).

**Figure 3:**
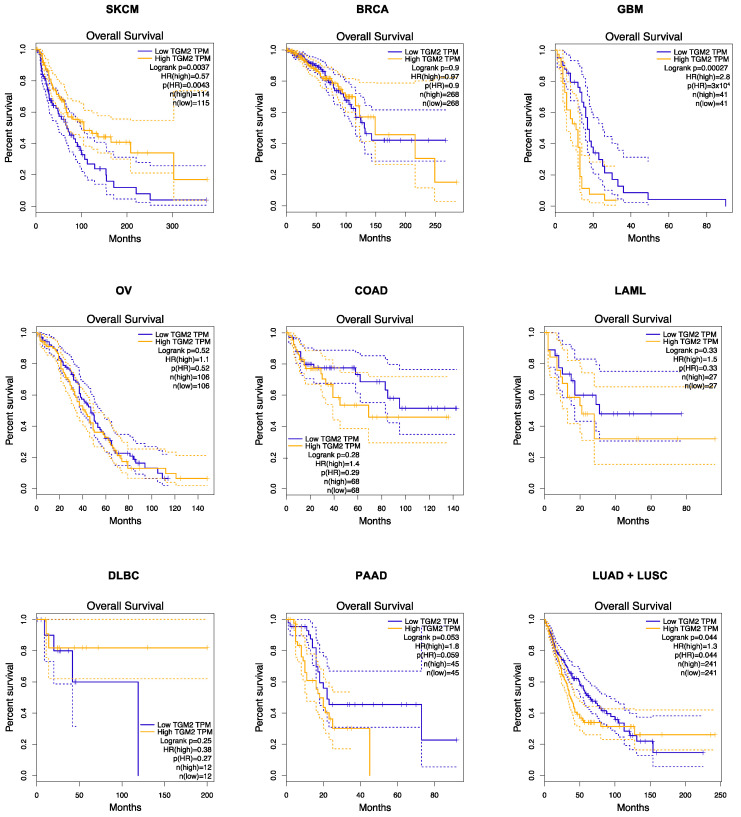
Overall survival based on *TGM2* expression level in SKCM (skin cutaneous melanoma), BRCA (breast invasive carcinoma), GBM (glioblastoma multiforme), OV (ovarian serous cystadenocarcinoma), COAD (colon adenocarcinoma), LAML (acute myeloid leukemia), DLBC (lymphoid neoplasm diffuse large b-cell lymphoma), PAAD (pancreatic adenocarcinoma), and LUAD + LUSC (lung adenocarcinoma + lung squamous cell carcinoma) was obtained through a Kaplan–Meier curve by sorting samples for high (yellow line) and low *TGM2* (blue line) expression groups according to quartile (high cutoff = 25%; low cutoff = 75%) on GEPIA. Percent survival was plotted, and p-values are shown as per figure specifications. The dotted lines represent the 95% Confidence Interval.

To better clarify the association between *TGM2* expression and overall survival in the same cohort of patients, we used a Kaplan–Meier curve by sorting samples for high and low levels of *TGM2* expression according to median *TGM2* mRNA levels. These analyses confirmed the prognostic value of TG2 in melanoma, glioblastoma, pancreatic adenocarcinoma, and lung carcinoma, as depicted in Figure 3.

**Figure 4:**
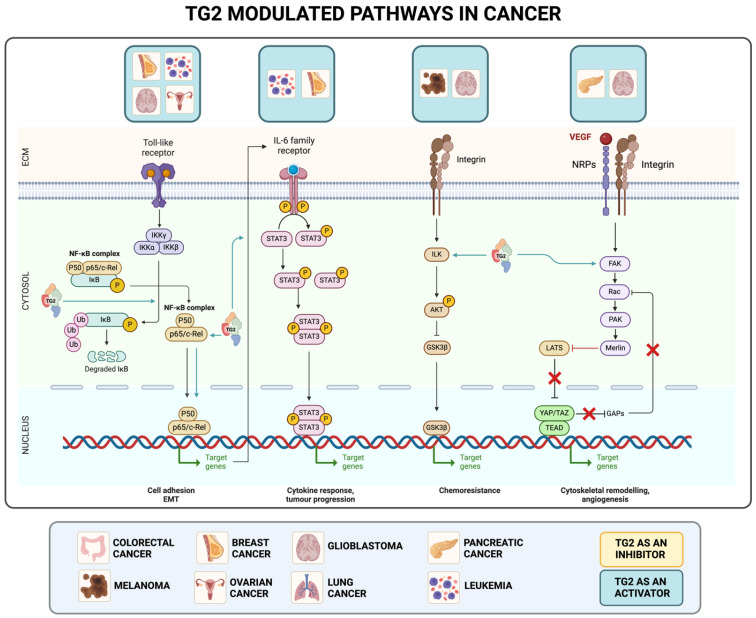

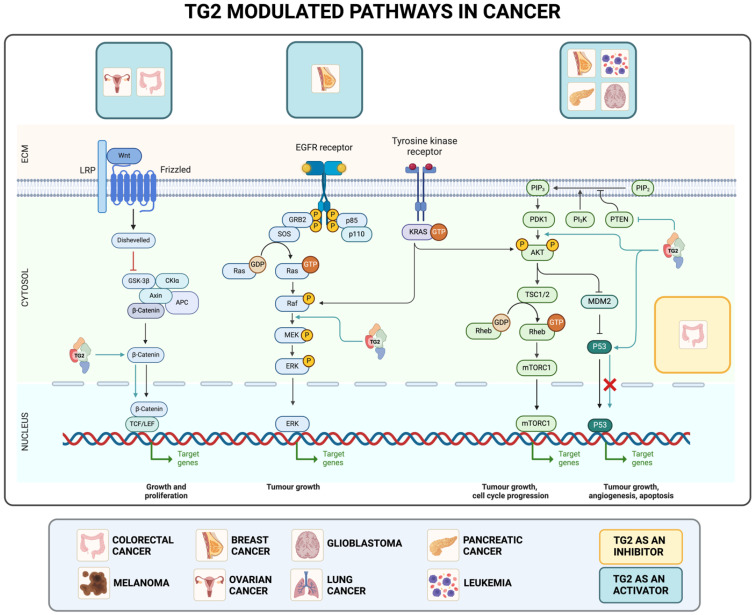
Schematic representation of TG2′s involvement in different pathways and tumors. For each axis, the following are indicated: TG2′s effect (orange TG2: inhibitor; blue TG2: activator), the tumor type in which this signaling is described, and its role in cancer progression. ECM: extracellular matrix.

TG2 plays a pivotal role in various cancer-related processes and pathways (Figure 4), contributing to several cancer hallmarks [17,44].

For instance, TG2 promotes uncontrolled cellular proliferation by interacting with TGF-β, a cytokine involved in proliferation, differentiation, and immune function. TGF-β regulates TG2 through a direct interaction with NF-κB on its IKB regulatory subunit and leads to cell proliferation. This process consequently contributes to the formation of spheroids and promotes metastasis [44]. It has also been demonstrated that TG2 leads to the accumulation of β-catenin, which translocates into the nucleus and activates the expression of CyclinD-1 and c-Myc, enhancing cell proliferation [17,24]. TG2 also mediates the regulation of the extracellular signal-regulated protein kinase 1 and 2 (ERK1/2) pathways that promote proliferation as well.

The ability to proliferate is related to the evasion of regulatory processes in cancer that are often managed via onco-suppressive pathways. With this concern, TG2 has been recognized to modulate two fundamental onco-suppressors: p53 and Rb. However, TG2′s kinase activity can also impair the negative feedback loop that involves MDM2 (Mouse Double Minute 2 protein) and p53; p53 induces MDM2 transcription, and MDM2 ubiquitinates p53 to induce its degradation. In this way, TG2-phosphorylated p53 stays undegraded as MDM2 does not ubiquitinate it and it accumulates, promoting apoptotic events [45].

Increased TG2 levels inside eukaryotic cells are considered a marker of apoptosis. However, deepening the relationship between TG2 and apoptosis, it was shown that TG2′s pro-apoptotic or anti-apoptotic effects were based on cellular context and changes in structural conformation (further influenced by Ca2^+^ and GTP concentrations). In this regard, it was demonstrated that TG2′s crosslinking of Sp1 induces apoptosis, while its action on caspase-3 and Bax can inhibit programmed cell death [44,46,47].

Cell death is prevented in cancer by a process called the epithelial to mesenchymal transition (EMT). The EMT consists of a phenotypic switch which includes the downregulation of adhesion molecules and the contemporary reprogramming of gene expression toward a mesenchymal profile with which survival factors are overproduced. This transition is often adopted by cancer cells to avoid death during the metastatic spreading phase [48,49]. TG2 also helps cells that undergo the EMT to acquire further stemness and plasticity, contributing to support stemness not only in primary tumors but also in their metastases [50,51]. TG2′s activity is indeed linked with the formation of cancer stem cells (CSCs) as it increases, through a non-canonical NF-κB pathway, the expression of CD44, their typical marker and a promoter of immortality, metastasis, chemoresistance, and a stem-like phenotype [44,52]. This is also explainable through integrin clustering that triggers intracellular growth and survival signaling pathways (PI3K/AKT, Hippo, and YAP (Yes-Associated Protein) and TAZ (transcriptional coactivator with PDZ-binding motif) [32,53].

Even ECM homeostasis is regulated by TG2 under normal conditions. In particular, ECM permeability and integrity are controlled thanks to the balance between TG2 (which regulates ECM stiffness) and metalloproteases (MMPs, which degrade ECM components). MMPs promote new matrix synthesis by fibroblasts, which then secrete TG2 and LOXs to adjust its consistency. A metalloprotease’s physiological role is to degrade ECM proteins, thus opposing TG2 crosslinking and stabilization [54,55].

TG2 also activates the FAK pathway to increase the contractility of tumor cells and the stabilization of focal adhesions. Its crosslinking activity, exerted on integrins and fibronectin, enhances adhesion, cell attachment, and invasion [56,57,58]. Particularly, in melanoma models, TG2 has been proven to stabilize contacts between circulating tumor cells and the subendothelial matrix but also to downregulate metastasizing capacity [59].

In the following paragraphs, we will discuss the different roles of TG2 in several cancer types, briefly reporting what has been discovered so far and highlighting recent discoveries to finally identify new possible therapeutic approaches involving TG2 in the treatment of different tumors.

### 2.1. TG2 and Melanoma

**Figure 5:**
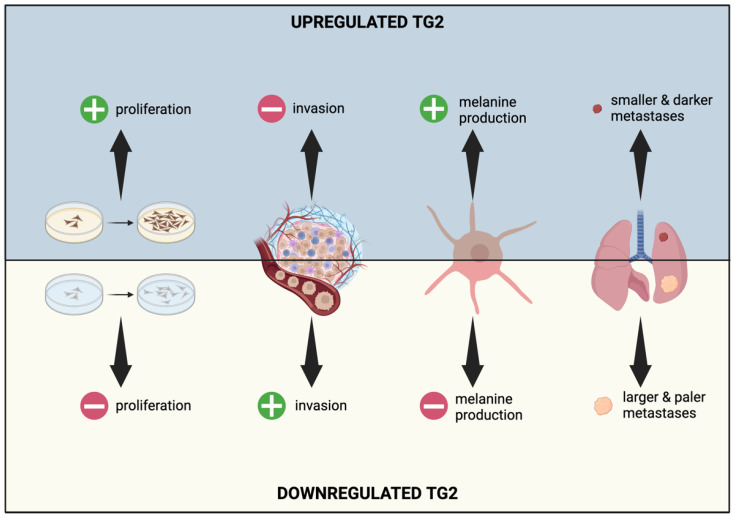
Representation of TG2 upregulation and downregulation effects in SKCM. Different levels of TG2 expression modify the characteristics of proliferation, invasiveness, melanin production, and metastasis formation in melanoma cells.

Skin cutaneous melanoma (SKCM) is a highly aggressive cancer with a 5-year survival rate lower than 5% in stage III or IV of the disease. The ability of SKCM to form metastases and spread represents a challenge which remains unaddressed [60]. Unlike normal cells, the TG2 expression level is upregulated in metastatic melanoma, correlating with an invasive stage of the disease [20,61], and promotes apoptosis in neoplastic cells (Figure 5) [62].

Metastases development is a critical issue in cancer treatment. In melanoma cells, TG2 seems to have a key role in the stabilization of the ECM and in the inhibition of tumor cell migration. This confirms TG2 as a positive prognostic factor, with its absence fundamental for metastases growth [61]. Furthermore, host tissues appear to respond to tumor invasion by increasing TG2 expression. In other types of tumors, the shift to a highly invasive phenotype is accompanied with an increase in TG2 expression and/or activity [61].

Cell motility is a critical step important to supporting the formation of metastases. In 2008, Kim and colleagues demonstrated that TG2 production is supported by hyaluronic acid through the nuclear factor kappa-light-chain-enhancer of activated B cell (NF-κB), Rac Family Small GTPase 1 (RAC1), and the Focal Adhesion Kinase (FAK). Thus, TG2 inhibitors can decrease the cellular motility of melanoma cells [57].

We recently demonstrated a new mechanism involving TG2 in melanoma metastasis. We showed the existence of a microphthalmia-associated transcription factor (MITF)–TG2 axis involved in the phenotype switching of SKCM cells [20]. In particular, we observed that high levels of TG2 correlate with high MITF levels and an increased capacity to enter into the nucleus to act as a transcription factor, finally activating several genes involved in the pigmentation process and in leading to a differentiated/melanocytic and less invasive phenotype. Conversely, low levels of TG2 impact both MITF expression and nuclear translocation, supporting an undifferentiated and more invasive/mesenchymal phenotype [35]. Thus, TG2 KO melanoma cells are less differentiated, unable to pigment, and form larger metastases when injected in vivo [57]. Moreover, TG2 is a positive prognostic factor as its knock-out (KO) seems to be correlated with the impairment of the immune cell recruitment and activation [43].

In addition to the formation of metastasis, resistance to chemotherapy is also a critical problem in SKCM treatment. Dacarbazine is one of the main drugs for melanoma treatment [6]. Tumor cells that express lower levels of TG2 are more sensitive to treatment with this compound, showing a clear correlation between a high level of expression of TG2 and resistance to chemotherapy. It has also been demonstrated that TG2 expression activates integrin signaling pathways to enhance melanoma cell chemoresistance [62].

Tumor growth is frequently related to an increase in extracellular matrix production. In SKCM, the G-protein coupled receptor GPR56, which binds the extracellular matrix (ECM), interacts with TG2, suppressing the development of the tumor. GPR56 and TG2 have opposite roles in melanoma: while TG2 promotes melanoma growth, GPR56 internalizes TG2, reducing its effect [63].

### 2.2. TG2 and Breast Cancer

**Figure 6:**
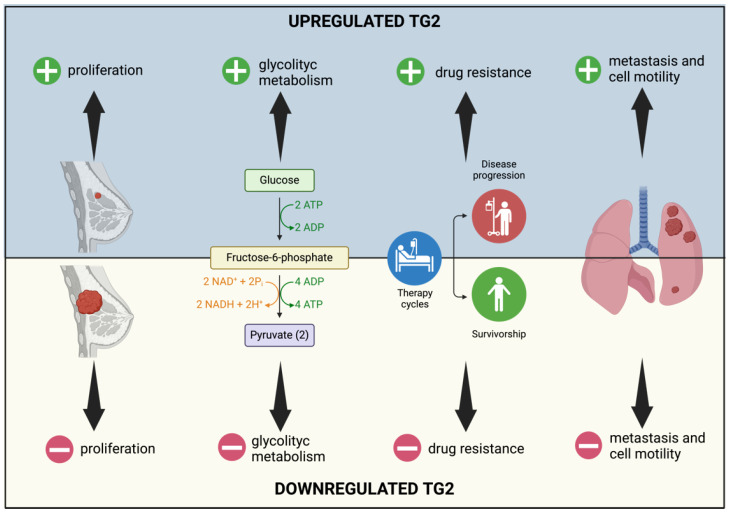
Representation of the effects of TG2 upregulation and downregulation in breast cancer. Different levels of expression of TG2 modify the proliferation capability, the glycolytic metabolism, drug resistance, and the ability to form metastases.

Breast cancer (BC) stands as the prevailing malignant tumor among women and is the second most common cause of cancer-related deaths in the Western world. Despite significant efforts in recent decades, the mechanism driving tumorigenesis and progression remains elusive [64]. Since 1996, it has been postulated that TG2 plays a role in breast cancer [26], demonstrating its involvement in fostering the EMT [48], advancing metastatic progression [65,66,67], and contributing to drug resistance [36,37,38]; see Figure 6.

TG2 has been also evaluated as a promising breast cancer prognostic biomarker that sustains cell proliferation and glycolytic metabolism through the mitogen-activated protein kinase/extracellular signal-related protein kinase/lactate dehydrogenase (MEK/ERK/LDH) pathway or by inducing hypoxia-inducible factor 1-alpha (HIF-1α) via NF-κB [64].

Moreover, a correlation between breast cancer cell motility and TG2 expression and localization has been demonstrated: exposure to doxorubicin, a chemotherapeutic drug, increased TG2 levels, thus triggering the EMT and supporting cell motility due to an interaction between TG2 and vimentin. On the other hand, treatment with NC9, an irreversible TG2 inhibitor, altered the subcellular distribution of TG2 and its colocalization with vimentin, inducing the nuclear accumulation of TG2 and consequent gene expression modification [48]. Additionally, the GTP-binding activity of TG2 promotes the EMT and the bone metastasis downregulation of miR-205, thus increasing the expression of the EMT marker ZEB1 [67].

Even in breast cancer, TG2 expression promotes the metastatic process: weakly migratory metastatic cells can release TG2-containing microvesicles, causing fibroblast activation and inducing tumor stiffening and spreading [65]. The role of TG2 in promoting the establishment of a pulmonary metastatic niche is further supported by another study which highlights the induction of fibronectin fibrillogenesis on the surface of TG2 micro-vesicles and a consequent reprogramming of lung fibroblasts [66].

A role of TG2 in promoting resistance to common anti-cancer drugs, including doxorubicin [36], neratinib [37], and PD-L1 inhibitors [38], has been shown. For example, TG2 mediates NF-κB activation, interleukin-6 (IL-6) upregulation, and Janus kinase/ signal transducer and activator of transcription 3 (JAK/STAT3) induction, thus promoting tumor progression, the acquisition of a stem-like phenotype, and neratinib resistance [37]. Another study suggests that TG2 could be considered a valuable predictive marker for identifying patients with triple-negative breast cancer who may be resistant to PD-L1 inhibitors. In fact, it was observed that TG2 induces phosphatase and tensin homolog (PTEN) and nuclear factor of kappa light polypeptide gene enhancer in B-cell inhibitor alpha (IκBα) proteasomal degradation, leading to phosphoinositide 3-kinases/protein kinase b (PI3K/AKT) and NF-κB activation, which is responsible for chemokine (C-C motif) ligand 2 (CCL2) and programmed death-ligand 1 (PD-L1) expression and, therefore, PD-L1 inhibitor resistance [38].

Finally, TG2 is activated by variations in intracellular Ca^2^/K^+^ due to the Kv10.1 voltage-dependent K channel. Kv10.1 is related to breast cancer cells’ ability to invade and metastasize. The combination of TG2 and Kv10.1 inhibitors could represent a novel therapeutic strategy [68].

### 2.3. TG2 and Glioblastoma

Glioblastoma (GBM) is one of the most aggressive glial tumors. A lack of targeted treatments results in extremely poor patient survival, with a 2-year survival rate barely reaching 10% [69]. So far, suggested therapies, span surgery, radiotherapy, and chemotherapy (mainly using Temozolomide), but despite this, a glioma stem cell population often causes resistance and relapse [70].

GBM can be further classified into four main groups: (1) proneural, (2) neural, (3) classical, and (4) mesenchymal, the subtype most resistant to conventional therapy [69]. It has been shown that a depletion of TG2 guarantees increased mouse survival thanks to a GBM size reduction and increased therapeutic efficacy. Accordingly, patients with high TG2 levels show a worse prognosis [69]. This is due to high TG2 levels in the tumor’s perinecrotic area which regulate key transcription factors, such as C/EBPb, TAZ, and STAT3, finally promoting GBM growth.

Radioresistant GBM also reveals higher TG2 and SDC-1 levels, suggesting the involvement of these two proteins in the regulation of the tumor’s aggressiveness. Their interaction promotes the fusion of autophagosomes with lysosomes in a process mediated by EPG5 and the subsequent translocation of TG2 into the lysosome, conferring typical radioresistance to GBM [71]. TG2 was also identified as a possible promoter of GBM cell proliferation, even if the full mechanism still needs to be clarified [72]. In addition, only some GBM subtypes respond to TG2 inhibitors, showing once more the controversial and gene-specific role of this protein [72].

Regarding cell survival, TG2 promotes uncontrolled cell promotion, acting on the Akt and NF-κB pathways [73].

### 2.4. TG2 and Ovarian Cancer

Epithelial ovarian cancer (EOC) develops from the epithelial layer covering the ovaries; the preferred therapy for this neoplasm is surgical resection followed by consolidative platinum-based chemotherapy in combination with Paclitaxel. Despite a good response to therapy, patients frequently relapse, bringing the 5-year survival rate down to 30% [74].

TG2 is known to be overexpressed in EOC, and its presence enhances peritoneal metastatization as it is required for consistent dissemination and adhesion to the peritoneal matrix [75]. The mechanism underlying this invasive capacity depends on the link between TG2 and Matrix Metalloproteinase-2 (MMP-2), which ends up being transcriptionally regulated. In fact, it was reported that TG2 binds and leads to the degradation of Protein Phosphatase 2 (PP2A), which normally suppresses the cAMP response element-binding protein (CREB). Consequently, CREB can bind the promoter of the MMP-2 gene, enhancing its transcription [75].

TG2 can also interact with integrin-β1 and fibronectin, creating a ternary complex at the plasma membrane. It has been demonstrated that the upregulation of these three proteins in ovarian cancer stem cells (OCSCs), and their molecular targeting, can disrupt the stem-like phenotype by dampening the Wnt/β-catenin signaling cascade. In the complex, TG2 can indeed interact with Frizzled (Fzd7), a Wnt ligand receptor, triggering OCSC proliferation and tumorigenicity [76]. In addition, it has been described how TG2′s catalytic activity guarantees an interplay with key transcription factors implied in Wnt/β-catenin pathway modulation [20].

In addition, TG2 regulates other important signaling pathways in EOC, such as the NF-κB cascade which is sustained by TG2′s sequestration of the inhibitor IκBα, ultimately leading to better tumor cell adhesion [75]. Moreover, extracellular TG2 induces the EMT by activating noncanonical NF-κB signaling, overall presenting TG2 as a metastasis enhancer at the cell–matrix interface [77]. TG2-mediated phosphorylation and complexation occurs also with Integrin-Linked Kinase (ILK). This event has been shown to correlate with a worse survival rate in patients [78].

Regarding tissues near tumors, a study demonstrates how TG2 promotes delayed OC growth by enhancing the recruitment of CD8^+^ T cells and the loss of immunosuppressive myeloid cell populations, highlighting once again the cell/tissue/context-specific role of the protein [79].

Finally, it has been recently shown that high Ca^2+^ levels induced by high-glucose conditions lead to ROS production and TG2 activation, with a consequent disruption of cell-to-cell contacts and ovarian cancer cell migration. Interestingly, human C-peptide is able to inhibit these high-glucose effects allowing us to hypothesize a potential therapeutic use for it [80].

### 2.5. TG2 and Colorectal Cancer

According to the World Cancer Research Fund International, colorectal cancer (CRC) is the third most frequent cancer worldwide in men and the second most frequent cancer in women [81].

**Figure 7:**
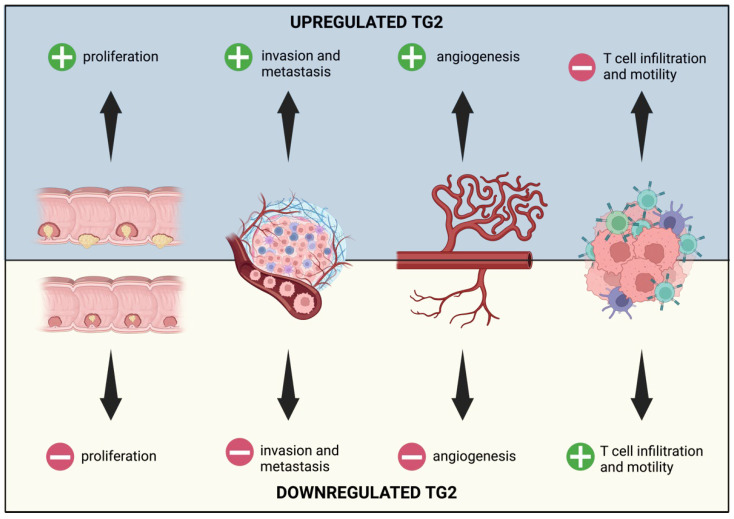
Representation of the effects of TG2 upregulation and downregulation in colorectal cancer. Different levels of expression of TG2 modify proliferation, invasion, angiogenesis, and metastasis capabilities. Moreover, TG2 overexpression correlates with reduced T cell ability to infiltrate and move.

TG2 plays a significant role in CRC development and progression. TG2 KO leads to a decrease in cell viability in vitro and a reduction in tumor development in vivo [82]. Indeed, TG2 is upregulated in patient tumoral tissues compared to healthy ones and is associated with poor prognosis and a reduced survival rate [83]. Similar to samples from patients, TG2 upregulation was also observed in CRC cell lines (e.g., HCT-116 and LoVo) [83]. In addition, Fernández-Aceñero et al. correlate the expression of TG2 in the stroma, which is required in CRC metastatic progression, with a high risk of relapse, while its epithelial expression was associated with poor overall survival [84]. RNA interference studies indicate that TG2 downregulation impairs cell viability, angiogenesis, and induces apoptosis through Caspase-3 upregulation [83]. In particular, TG2 expression promotes nuclear accumulation of β-catenin, while its inhibition reduces β-catenin expression and ERK1/2 activation [85], Figure 7.

Furthermore, recent studies demonstrated a role for TG2 in the inactivation of p53. In fact, TG2 knockdown also determined modifications in gene expression, such as the downregulation of pathways that involve NF-κB, KRAS, inflammatory and hypoxia mediators, estrogens, and the upregulation of p53 signaling. A proximity ligation assay proved a physical interaction between p53 and TG2 which has the potential to influence p53 function and activate the Caspase-3 pathway. Moreover, an elevated level of p53 phosphorylation has been observed in tumors that exhibit low TG2 expression, corroborating evidence for the pro-apoptotic effect of TG2 knockdown [82]. It has been demonstrated that there is a correlation between the presence of p53 mutations and the level of TG2 expression as tumors exhibiting p53 mutations show a greater level of TG2 expression. The inhibition of TG2 results in the induction of p53-mediated apoptosis, which in turn reduces cancer cell proliferation. For this reason, TG2 can represent a therapeutic target in CRC [86].

It has been demonstrated that TG2 is regulated by ETS1, and both these proteins are targeted by miR-532-3p, which is downregulated in CRC and can restrain the overexpression of β-catenin. As a result, miR-532-3p exerts an inhibitory effect on CRC progression, promoting chemosensitivity and activating p53 [87].

Further studies have found an association between TG2 expression and the metastatic capabilities of CRC. It appears that the knockdown of TG2 can influence the upregulation of E-cadherin and the downregulation of N-cadherin and vimentin, thereby altering the metastatic potential of cancer cells in terms of invasion and migration. It is noteworthy that actin appears to be an intracellular substrate for TG2, indicating the potential ability of TG2 to influence cytoskeleton remodeling during cell motility [87].

Enhanced expression of TG2 is associated with a higher level of self-renewal capability, whereas its elimination leads to a decrease in it. This phenomenon may be attributed to the fact that stemness-associated proteins, such as CD133 and Sox-2, were increased in cells exhibiting elevated TG2 expression [88].

TG2 is also involved in the inhibition of T cell infiltration and motility in the tumor microenvironment. It appears that TG2 is necessary for the formation of a coating of CXCL12-KRT-19: TG2 binds KRT-19 in the cytosol, and the complex is secreted in the extracellular space where CXCL12 performs a nucleophilic attack. When the complex is formed, it can self-assemble with KRT-8, forming a coating around cancer cells. This coating is able to cross-link CXCR4 on nearby T-cells’ surfaces, suppressing the T cells’ motility [89].

### 2.6. TG2 and Leukemia

Leukemia is cancer of blood-forming tissues, including bone marrow and the lymphatic system. The word “leukemia” refers to a variety of pathologies with different characteristics: acute or chronic leukemias and myeloid, histiocytic/dendritic, and lymphoid neoplasms [90,91].

In the field of the acute leukemias, TG2 is involved in blast motility [40]. TG2 expression, which is higher in relapsed leukemia with respect to the level at diagnosis, positively correlates with several adhesion proteins (e.g., fibronectin, FAK, etc.) [92].

TG2 is important in acute promyelocytic leukemia (APL), which is characterized by the accumulation of immature granulocytes called promyelocytes [93]. When it is not rapidly diagnosed and treated, APL is considered one of the worst leukemias [93]. In the context of canonical APL treatment with all-trans retinoic acid (ATRA) and arsenic trioxide (ATO), TG2 expression is increased, and this leads to an intensification of inflammation through the NF-κB pathway [94]. Consequently, TG2 inhibition reduces ROS production, inflammation processes, and consequent organ damage [95]. To turn off the lethal effects of the inflammatory response (called differentiation syndrome, it is typically present after ATRA-ATO treatment [96]) is crucial to improving patient outcome [94]. Moreover, Jambrovics K et al. highlight the mechanism through which TG2 is able to extend APL cell survival following conventional ATRA-ATO treatment. They described the role of TG2 in signalosome platform initiation. This, in turn, triggers the hyperactivation of downstream mTORC2-AKT signaling, and it consequently leads to the phosphorylation and subsequent inhibition of FOXO3, a critical pro-apoptotic transcription factor [97].

A role for TG2 has also been described in T- cell lymphoblastic leukemia (T-ALL). T-ALL is an aggressive hematologic neoplasm characterized by the accumulation of early T cell progenitors in the bone marrow [98]. Jung H et al. described that T-ALL blasts highly express TG2 and that the inhibition of this protein reduces cell resistance to steroids in association with decreased NF-κB activity [99]. In T cell lymphoblastic lymphoma, a subtype of T-ALL characterized by the accumulation of T-blasts in lymph nodes [100], the modulation of IL-6/JAK/STAT3 by the siRNA inhibition of TG2 has been demonstrated. The impairment of these signaling pathways leads to a reduction in T cell lymphoma cell proliferation [101].

TG2 is also important in Mantle Cell Lymphoma (MCL), which is an incurable lymphoma originating from the mantle zone of the lymph node (where memory and naïve B cells and T cells can be found) [102]. In this neoplasm, TG2 expression leads to the constitutive activation of NF-κB and consequent chemoresistance [103]. Moreover, TG2 can increase IL-6 production, triggering autophagy through the JAK/STAT3 axis to promote MCL cell survival [103].

TG2 can play a relevant role in Chronic Myeloid Leukemia (CML). In CML, myeloid cells grow uncontrolled in bone marrow and accumulate in the blood; in both these districts, there is a proliferation of mature granulocytes (neutrophils, eosinophils, and basophils) [104]. Kang S et al. demonstrate that TG2 is recruited at the plasma membrane during erythroid differentiation and that the impairment of TG2 expression in a CML-K562 cell line can lead to a delay in differentiation through the PI3K/Akt pathway [105]. Moreover, Ha et al. hypothesized a crucial role of TG2 recruitment on the cell membrane, thanks to the ganglioside GD3/α1-AR-mediated signaling pathway, for CML-K562 cell erythroid differentiation. This evidence suggests a promising solution for leukemia treatment, providing a rationale for a combination therapy between a GD3/α1-AR/TG2 axis inhibitor and Imatinib or another routinely used drug [106]. Finally, it has been demonstrated that TG2′s active form is needed for caffeic acid-induced apoptosis. In particular, TG2 inhibitors are able to reduce phosphatidylserine extroversion and caspase activation. Consequently, TG2′s pharmacological activation could represent a possible strategy to enhance to enhance available therapies [107].

### 2.7. TG2 and Pancreatic Cancer

Pancreatic cancer is the fourth most common cause of cancer death worldwide. Pancreatic ductal adenocarcinoma (PDAC) accounts for the majority of pancreatic malignancies, and most patients present with disseminated disease at diagnosis [108]. Higher TG2 expression levels in PanINs compared to normal pancreatic tissues and which increase with pancreatic cancer progression have been observed.

**Figure 8:**
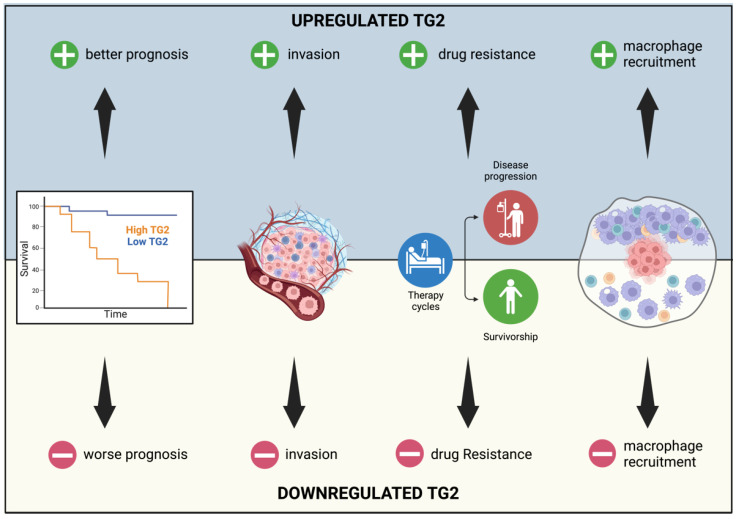
Representation of the effects of TG2 upregulation and downregulation in pancreatic cancer. Different expression levels of TG2 lead to a different prognosis and drug resistance. Moreover, TG2 modulation is correlated with cancer cells’ efficiency at invading tissues and recruiting macrophages.

Indeed, recent works proposed a role for TG2 in the pathogenesis of pancreatic cancer [109], contributing to chemotherapy resistance [110] and influencing immune infiltration [111]. More specifically, a comprehensive study conducted to understand the importance of transglutaminases in human cancers revealed that high TG2 levels in pancreatic cancer are associated with worse patient survival, resistance to gemcitabine, and an increase in macrophage recruitment due to the release of the chemokine CCL2 by cancer cells [111]; see Figure 8.

Additionally, two recent works highlighted how TG2 can stimulate cancer growth through the activation of YAP/TAZ transcription factors. Moreover, when TG2 is secreted by pancreatic cancer cells, it can activate cancer-associated fibroblasts to produce laminin A1 which, in turn, shields tumor cells from gemcitabine-induced cell cytotoxicity by triggering signaling pathways, such as the FAK pathway [59,110]. Another study supports a role for TG2 in regulating the FAK/AKT survival pathway due to the stimulation of PTEN proteasomal degradation after inhibiting its phosphorylation. This is responsible for influencing pancreatic cancer cells’ invasiveness and response to chemotherapy [112].

### 2.8. TG2 and Lung Cancer

Despite ranking second in incidence, lung cancer remains the leading cause of cancer-related death [113]. According to the 2021 World Health Organization (WHO) Classification of Thoracic Tumors, lung cancer encompasses a very heterogeneous group of approximately eighty different tumors characterized by distinctive morphology, immunohistochemistry, and molecular markers [113]. We can broadly distinguish between (1) non-small cell lung cancer (NSCLC), further categorized into lung adenocarcinoma (LUAD), lung squamous cell carcinoma (LUSC), and large-cell carcinoma (LCC); and (2) small cell lung cancer (SCLC), divided in two subtypes: oat cell cancer (OCC) and combined small cell carcinoma (C-SCLC). Concerning NSCLC, two in vivo studies on a patient cohort [113,114] suggested TG2 as a biomarker of increased invasion/migration and poor prognosis, highlighting significantly increased TG2 expression in lung cancer tissues compared to normal ones and showing a correlation between TG2 upregulation and shorter disease-free survival in the non-adenocarcinoma subtype in a Korean cohort [113] and in both the non-adenocarcinoma and adenocarcinoma cancer subtypes in Chinese patients [114,115]. In vitro studies provided the greatest part of our knowledge about TG2′s role in lung cancer, highlighting its involvement in cell invasion migration and drug sensitivity. In more detail, using a less invasive LUAG cell line, CL1-0, and its highly invasive counterpart, CL1-5, Lee et al. showed that TG2 promotes the migration and invasion of lung cancer cells through a mechanism independent from its transamidase activity [41]. Lei et al. showed that TG2 confers radioresistance in NSCLC adenocarcinoma cell lines, promoting DNA repair and directly interacting with TOP2. Both TG2 expression and its transglutaminase activity were found to be upregulated; using the RNA interfering strategy, it has been demonstrated that TG2 reduction results in oxidative stress, inducing p53-independent extrinsic and intrinsic apoptosis [116]. Even if TG2′s role in SCLC is poorly investigated, a recent study showed that in acquired prexasertib-resistant cell lines, H792LYR and GLC4LYR correlate with increased *TG2M* expression [39].

## 3. TG2 and Microenvironment

In the intricate interplay between a tumor and its surrounding microenvironment, TG2 has consistently played a distinctive role, positioning TG2 as a possible prognostic biomarker. The presence and activity of this enzyme have been extensively acknowledged in the stromal tissue surrounding the tumor but also in various cellular components of the TME, including endothelial cells, cancer-associated fibroblasts (CAFs), immune cells, and adipocytes [17]. Importantly, TG2 exhibits varying expression levels depending on the cell type and the stage of the disease [44]. Altogether, these findings point out the role of TG2 in modulating various biological and biomechanical processes.

### 3.1. TG2 and Angiogenesis

One of the hallmarks of cancer is angiogenesis, a process implicated in generating new blood vessels which is involved in supporting the immune escape and the formation of metastases [117,118,119,120]. Indeed, the presence of blood vessels inside the tumor is important or feeding cancer cells with oxygen and nutrients, and it is further sustained by the release of pro-angiogenic factors such as VEGF, bFGF, and PDGF by tumor cells and the tumor microenvironment (TME) [121]. Despite limited success in therapies targeting pathological angiogenesis [122], the identification of new possible targets, including TG2, is crucial for developing more effective anticancer approaches.

The relationship between TG2 and angiogenesis is debated since both pro- and an anti-angiogenic roles have been reported [44]. TG2 is expressed in endothelial cells in the cytosol [123,124], but most of its impact on angiogenesis is mediated by its presence in the extracellular matrix. With regard to the cytosol, TG2 interference by siRNA reduced endothelial cell quantity by supporting cell cycle arrest in the G1 phase, the induction of apoptosis, and decreased cell adhesion, even if this role is ascribable to the extracellular pool [125]. In the extracellular space, a role of matrix-bound TG2–syndecan-4 interactions in cell adhesion [126,127] and that TG2 colocalizes with β1 integrin molecules, particularly in focal at-cell adhesion points, have been demonstrated [127]. Furthermore, the inhibition of extracellular TG2 crosslinking activity or TG2 downregulation reduce angiogenesis both in vitro by impacting tubule branching, as well as in vivo, by weakening vasculature formation in chicken embryos [128]. From a molecular point of view, TG2 inhibition leads to diminished fibronectin deposition and cell migration, both necessary processes during tubule formation, by impairing the VEGF-mediated cascade and finally leading to reduced Akt and ERK1/2 signaling [128]. Additionally, NF-κB /HIF1α was reduced in endothelial cells following the inhibition of the GTP-binding activity of TG2, achieved through its interaction with the gastric cancer-targeting peptide GX1 [129,130]. Moreover, HIF1α has been shown to be triggered by TG2 activity in several cancers, such as in renal carcinoma where, in turn, it supported the increased expression of VEGF after p53 degradation [131]. Finally, TG2 has a negative effect on the von Hippel–Lindau (VHL) tumor suppressor protein through its cross-linking activity, favoring the release of HIF1α from degradation by proteasomes and increasing vascularization and invasiveness: CHOP-mediated TG2 downregulation disrupts this mechanism, suppressing kidney tumor growth and angiogenesis [132].

Conversely, as described above, high TG2 levels hamper angiogenesis both in vitro and in vivo, finally ceasing cancer development. Again, the supplementation of exogenous TG2 addition impedes angiogenesis in a dose-dependent manner, supporting an augmented accumulation of extracellular matrix protein, finally impacting tumor growth, tumor angiogenesis, and animal survival [133].

In addition to these effects mostly related to the capability of TG2 to modulate endothelial cell signaling and angiogenesis, TG2 is also involved in influencing the mechanical properties of vascular walls. TG2 contributes to matrix remodeling, affecting the mechanical properties of collagen fibers, and plays a role in tuning vascular stiffness [134].

### 3.2. TG2 and Cancer Associated Fibroblast (CAFs)

Fibroblasts are the main tumor-supportive cells found in the TME, and they normally sustain the synthesis and deposition of ECM components. In this context, TG2 plays several roles in supporting fibroblast–matrix interactions, finally impacting cell spreading and migration, the restructuring of the ECM, wound healing, and fibrosis. During cancer, TG2 is involved in promoting fibroblast de-differentiation to a more tumor-supporting and myofibroblastic-activated phenotype [17,44,135]. Part of these functions in CAFs can be sustained by the well-known interplay between TG2 and TGFβ; indeed, TGFβ can activate CAFs, and this event correlates with patient poor prognosis [136,137,138]. In colorectal cancer, TG2 has been shown to be upregulated in CAFs compared to normal fibroblasts [139]. In addition, TG2 expression was assessed in CAFs from other types of tumors; in hepatocellular carcinoma, it was associated with a CAF-driven EMT via the IL-6/IL-6R/STAT3 axis [140], while in pancreatic cancer, it correlates with a worse prognosis since the TG2 secreted by cancer cells supports matrix deposition and promotes neoplastic cell proliferation via the activation of the YAP/TAZ signaling pathway [59]. In addition, TG2-activated CAFs can secrete laminin 1, impacting pancreatic cancer cell sensitivity to gemcitabine treatment [110].

### 3.3. TG2 and Immune Cells

The interaction between immune cells and a tumor is a crucial event during cancer development and invasion. In addition, cancer cells can impair the immune response by acting directly on immune system populations, by enrolling cells with immunosuppressive activity, or by secreting factors able to recruit specific immune cell subpopulations (e.g., regulatory T lymphocytes, immature dendritic cells, TAMs, and myeloid-derived suppressor cells) that can inhibit the immune response [141]. In this scenario, TG2 is likewise involved in controlling the maturation of different immune cells [10]. Indeed, TG2 is constitutively expressed in monocytes and macrophages, and it is important for process like cell adhesion and extravasation, which are fundamental for monocytes to reach the site of inflammation [142]. In addition, TG2 expression increases during the differentiation of monocytes into macrophages [143] or dendritic cells (DCs), where TG2 mediates the maturation of antigen-presenting cells in response to LPS [144,145]. Furthermore, TG2 has been shown to be involved in several immunological processes ranging from DC-T cell interactions, which endorse the adaptive immune response [44,146], to regulating the macrophage phagocytosis of apoptotic and necrotic cells [147], or to the modulation of T cell proliferation [146]. Indeed, in the TME, TG2 expression has also been found in myeloid cells, T, and B cells [17]. TG2 has been identified as a marker for tumor-promoting M2 macrophages [17]. According to cancer in PDAC and lung squamous cell carcinoma, TG2 expression has been identified as immunosuppressive and associated with a poor clinical outcome since increased numbers of M2 macrophages and regulatory T cells have been observed, along with reduced numbers of pro-B and memory B cells [148]. In this setting, the molecular mechanism is based on PD-L1 regulation, following NF-κB and STAT3 signaling activation [148]. In gastric cancer, a high level of TG2 activity enhanced inflammation and tumor growth by recruiting macrophages to the tumor via the IL-1β-mediated induction of CCL2 and CXCL10 [149]. In breast cancer, the inhibition of TG2 can be used as a therapeutic strategy to overcome PD-L1 inhibitor resistance in PD-L1(+) TNBC patients since this reestablished T cell-dependent cytotoxicity by impeding the expression of both PD-L1 and CCL2 [38]. In ovarian cancer, a lack of TG2 delayed tumor dissemination by inducing, in TG2 KO mice, less TAM infiltration and decreased PD-L1 expression, as well as an increased infiltration by cytotoxic T cells and T effector/memory cells [79]. CD8^+^ and CD4^+^ T cells derived from TG2 KO mice exhibited altered STAT1/STAT3 signaling in response to IFN-γ, IL-6, and TGF-β [79]. In this line, the CD30-TG2 axis is an essential signaling pathway for memory Th cell generation [150]. Moreover, TG2 plays a role as a negative regulator in humoral immune responses by modulating the expression of B-lymphocyte-induced maturation protein-1 (Blimp-1) and activation-induced cytidine deaminase (AID) [151].

Finally, TG2 has been recently correlated with the activation of the cGAS-STING pathway, a cellular cytosolic double-stranded DNA sensor that produces large amounts of type I IFNs, allowing for an innate immune response to infections, inflammation, and cancer. Indeed, in tumor cells, the activation of the STING pathway may pose an obstacle to the progression of early neoplastic cells by upregulating type I IFNs or other inflammatory genes [152]. In this context, TG2 negatively regulates STING signaling and can reduce IFN I production by preventing the interaction of TBK1 with IRF3, suggesting TG2 as a novel target to modulate immune responses in cancer [153].

## 4. Conclusions

**Figure 9:**
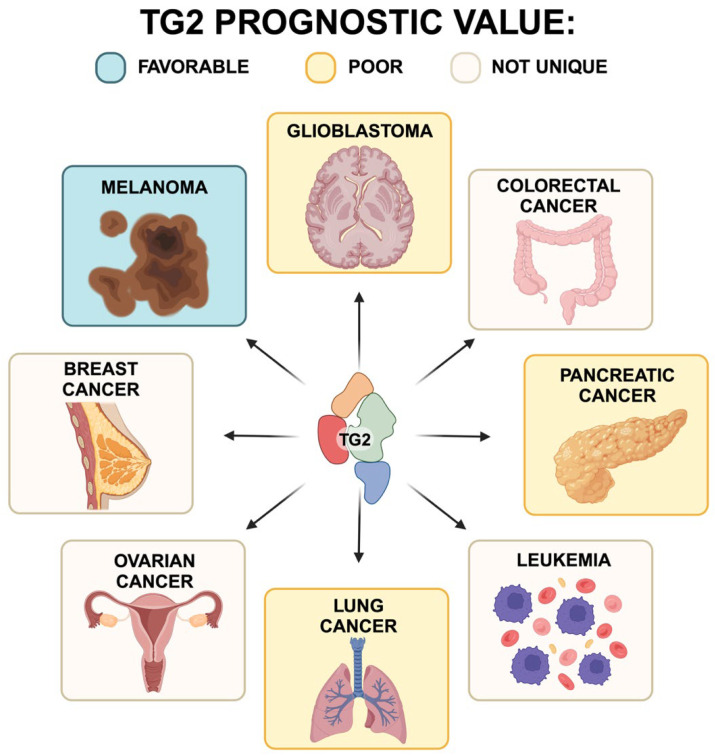
Prognostic significance of TG2 expression in different types of cancer. Blue: favorable; orange: poor; beige: not unique.

In this review, we have reported some of the most recent, updated literature concerning the role of transglutaminase type 2 in several types of cancers, as well its role in modulating and interacting with the tumor microenvironment. Altogether, the presented data clearly demonstrate an important function of TG2 in supporting several hallmarks of cancer, finally resulting in the favorable or a poor prognostic role of this enzyme (Figure 9).

Nevertheless, several aspects need to be clarified since TG2 has shown a controversial behavior, it is clear that targeting TG2 multiple activities inside and outside cancer cells and could finally bring us to a possible cure for several of the worst tumors.

## Figures and Tables

**Table 1:** Drugs for which TG2 is involved in resistance mechanisms.

Tumor Type	Drug	Reference
Skin Cutaneous Melanoma	Dacarbazine	[35]
Breast Cancer	Doxorubicin	[36]
	Neratinib	[37]
	PD-L1 inhibitors	[38]
Lung Cancer	Prexasertib Doxorubicin	[39]
		[40]

## Data Availability

Not applicable.
